# Edoxaban Improved End-QRS Notches and Early Repolarizations

**DOI:** 10.7759/cureus.62962

**Published:** 2024-06-23

**Authors:** Hidekazu Takeuchi

**Affiliations:** 1 Internal Medicine: Cardiology, Takeuchi Naika Clinic, Ogachi-Gun, JPN

**Keywords:** cardiac ct, transesophageal echocardiogram, lef atrium diverticula, early repolarization, end-qrs notch, edoxaban

## Abstract

Cardiac computed tomography (CT) images sometimes show a donut-like oval structure on the antero-superior wall of the left atrium (LA). What is a donut? Left atrium diverticula (LADs) are common, but there are many unknown features of LADs. The direct effects of pulmonary vein thrombi (PVTs) on the heart are poorly understood.

Herein, we report a case report in which we describe the different effects of edoxaban on LA thrombi, the LAD, coronary artery collaterals, early repolarizations, and end-QRS notches using cardiac CT and transesophageal echocardiography (TEE). First, we showed that there was a LAD on the anterior wall of the LA where the LA thrombi from the right lower pulmonary vein (RLPV) thrombi were connected.

To our knowledge, this is the first report to reveal LAD’s annular transformation and the beneficial effect of edoxaban on the end-QRS notch.

## Introduction

Research on retrieved thrombi has shown that the thrombi have calcified regions [1,2], suggesting that the thrombi are old. Pulmonary vein thrombi (PVTs) have no intrinsic clinical conditions; however, PVTs are common [3,4] and are currently underdiagnosed. PVTs have the potential to be the source of retrieved thrombi, indicating that PVTs can cause ischemic stroke (IS) and acute myocardial infarction (AMI) [5,6]. We have reported several cases of PVTs using cardiac computed tomography (CT) and transesophageal echocardiography (TEE) [3,4,7,8].

A previous study reported that 35 out of 57 (61%) elderly individuals with chest pain had PVTs [4] and that 17 of 35 (49%) patients with PVT had deployed thrombi in the left atrium (LA) [9], which were assessed using cardiac CT.

Recently, we reported not only several cases of PVTs but also several cases of LA thrombi deployed from PVTs using TEE that could not be identified using cardiac CT in many cases [7,8]. Thus, it is possible that PVTs are more common than previously thought. PVTs can cause IS and AMI by emitting larger particles. Additionally, PVTs can release small particles, such as neutrophil extracellular traps (NETs). NETs are known to be associated with many diseases, including type 2 diabetes mellitus (T2DM) [10], atherosclerosis [11], acute coronary infarction [12], and heart failure [13].

Currently, our knowledge of PVTs is limited. A better understanding of PVTs has the potential to improve treatment strategies and decrease potential distress. We report herein another case of PVT that was identified using cardiac CT and TEE.

Moreover, in several patients, PVTs were connected to the LA anterior wall between the ostia of the left upper pulmonary vein (LUPV) and the right upper pulmonary vein (RUPV). Left atrium diverticula (LADs) are often discovered around attachment regions [7,8].

LADs are common and have an incidence of 10~50%. LADs were discovered relatively recently, but their clinical meaning remains unknown. LADs are commonly located on the anterosuperior wall of the LA [14].

When a patient complains of chest pain, early repolarization (ER) and an end-QRS notch on an electrocardiogram (ECG) are important for assessing AMI. Moreover, the ER and end-QRS notch are associated with sudden cardiac arrest [15] and atrial fibrillation [16]. How to treat these patients is poorly understood.

In this case report, we reported that a patient with PVTs had LADs, an ER, and an end-QRS notch. Edoxaban's effects on these issues are unclear.

## Case presentation

A 70-year-old female with dizziness was examined using cardiac CT and TEE to assess PVTs. The dizziness was not as severe, and she felt it only a few times. No ameliorating factors or related symptoms of headache, nausea, vomiting, tinnitus, neck stiffness, or altered level of consciousness were recorded. She had no symptoms of chest pain or shortness of breath. Her physical examination was unremarkable, and her vital signs were within normal limits. Her body mass index (BMI) was 25.1 kg/m2. She had no history of AMI or IS. No previous treatment with warfarin or direct oral anticoagulants (DOACs) had been performed.

**Figure 1:**
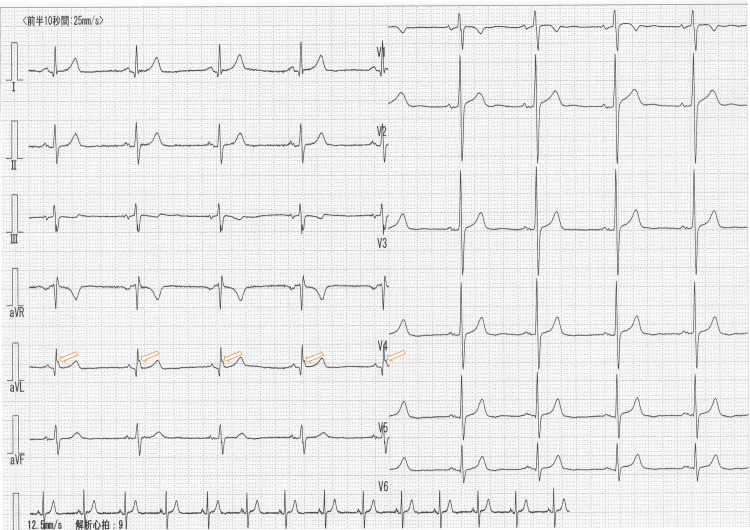
Arrows indicate end-QRS notches and early repolarization (ER)

ECG indicated sinus rhythm and an end-QRS notch in the aVL (Figure 1), and the patient’s heart rate was 54 beats/min. The serum D-dimer level was 0.6 μg/ml (normal; < 1.0 μg/ml), the activity of protein S was 149% (normal; 74-132%), and the activity of protein C was 82% (normal; 64-135%). The concentration of homosistein was 7.5 nmol/mL (normal: 5-15 nmol/mL).

**Figure 2:**
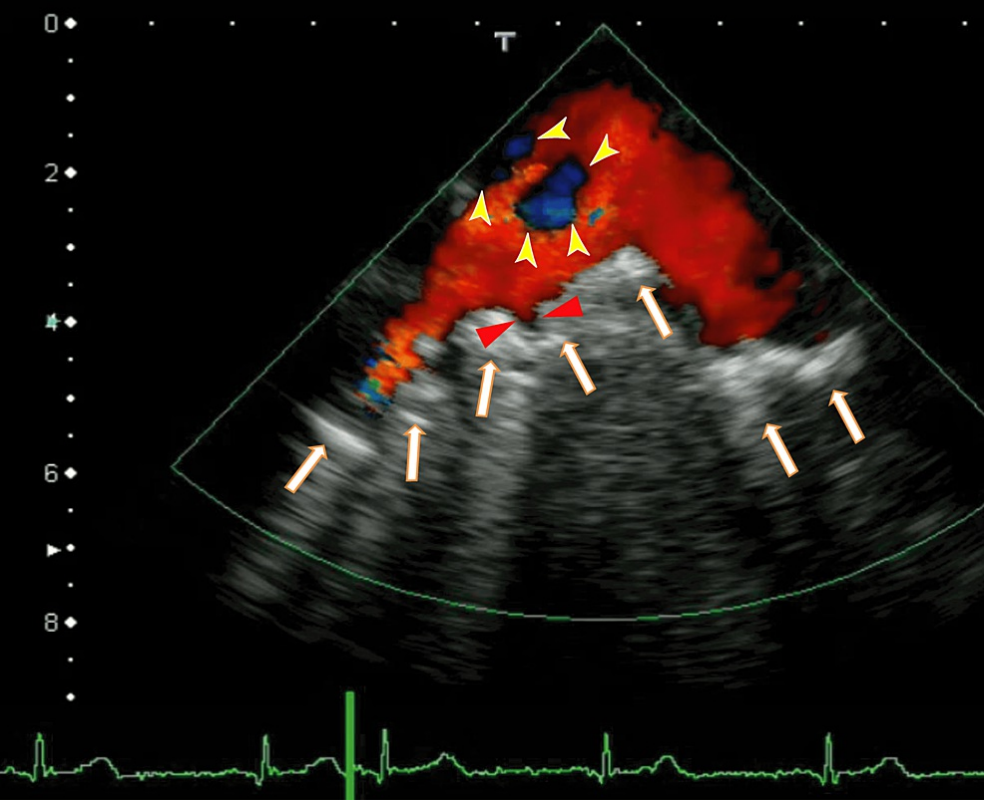
Transesophageal echocardiography (TEE) showing thrombi in the left atrium (LA) The thrombi were located between the ostia of the left upper pulmonary vein (LUPV) and the ostia of the right upper pulmonary vein (RUPV), and the location was the anterior wall of the LA (arrows). Blood influx from the LUPV and RUPV around the thrombus is shown in red. Dark thrombi were identified as a lack of red blood flow from the RUPV (yellow arrowheads). The left atrium diverticulum (LAD) can be identified (red arrowheads). LA: left atrium; LUPV: left upper pulmonary vein; RUPV: right upper pulmonary vein

The TEE revealed white thrombi in the LA between the left upper pulmonary vein (LUPV) and the right upper pulmonary vein (RUPV). There were rather vague whitish thrombi near the ostia of the right lower pulmonary vein (RLPV), which could be detected as a lack of red color (Figure 2). The video images demonstrated that the thrombus was moving like a frog-mouth in the LA between the LUPV and the RUPV, which connected to the anterior wall of the LA (Video 1). The right end of the LA thrombi seemed to be connected to the thrombi from the RLPV. We detected the LAD, an end-QRS notch, and the ER on her ECG.

**Video 1:** The video in Figure 2 Periodic movement can be easily understood. ER and an end-QRS notch could be observed on the ECG.

**Figure 3:**
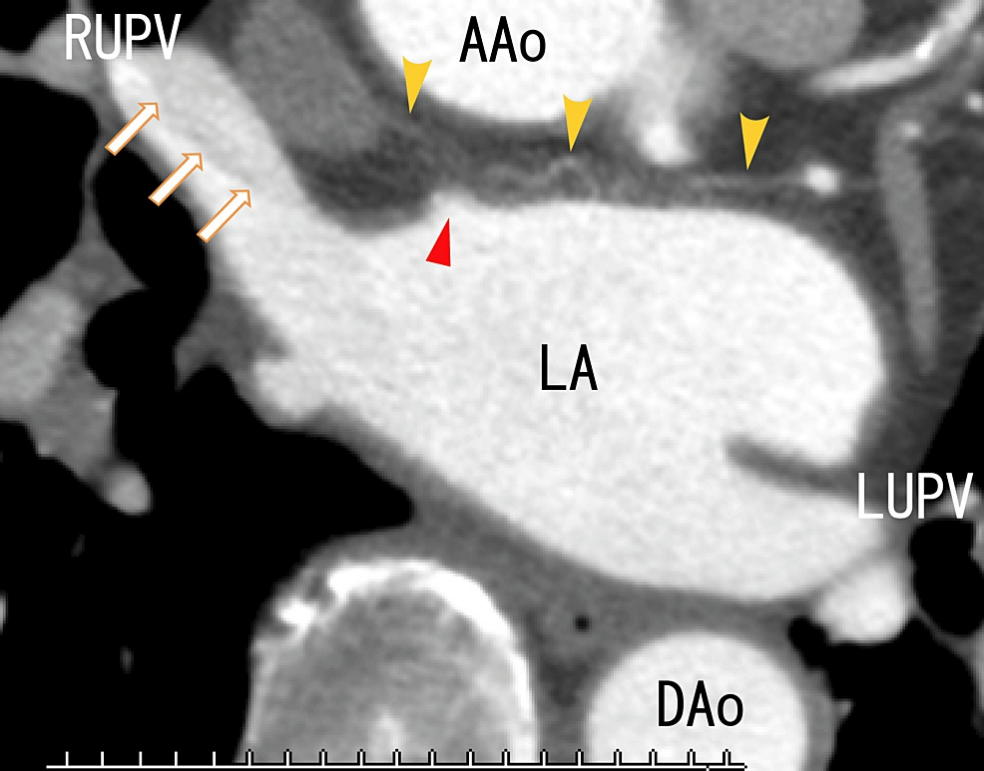
Axial 80-slice multidetector computed tomography (80-MDCT) images Axial 80-MDCT images show thrombi with clear margins in the RUPV (arrows). The LAD can be identified (red arrowhead). Collateral to the right atrium from the left circumflex artery (LCX) can vaguely be detected over the top of the LAD (yellow arrowheads). AAo: ascending aorta; DAo: descending aorta; LA: left atrium; LUPV: left upper pulmonary vein; RUPV: right upper pulmonary vein

**Figure 4:**
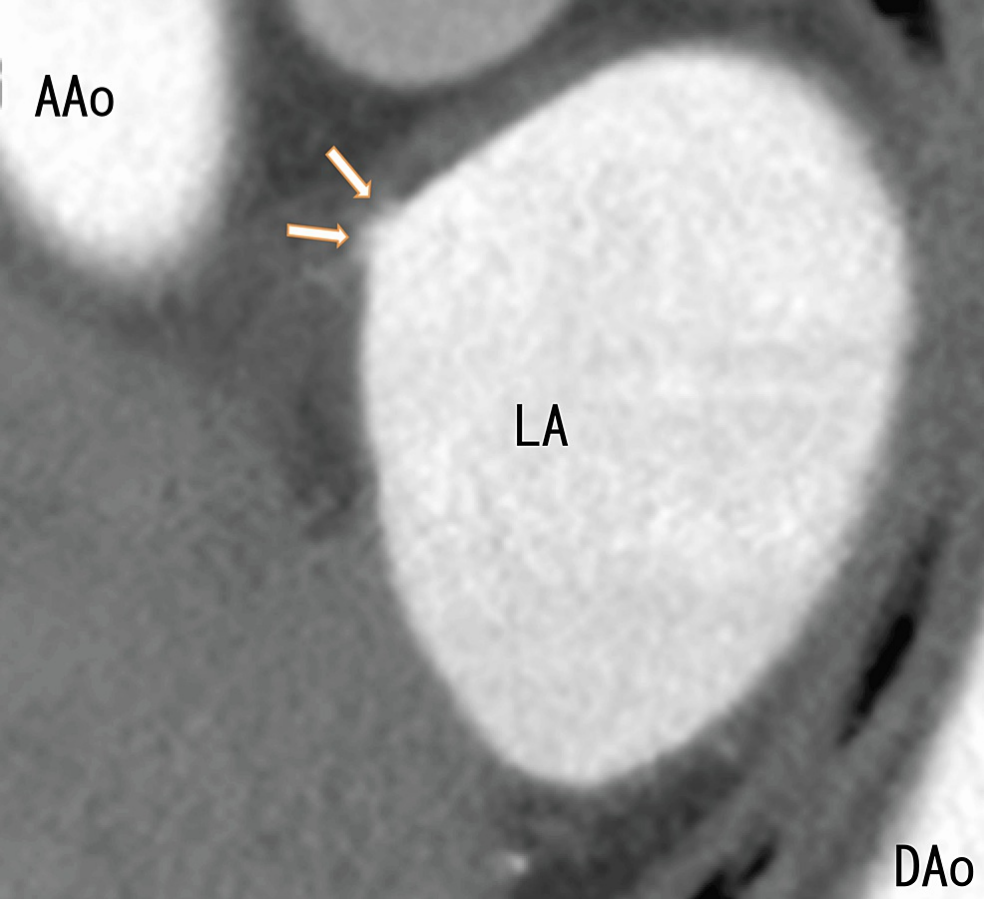
Sagittal images of 80-MDCT showing LADs on the anterior wall of the LA AAo: ascending aorta; DAo: descending aorta; LA: left atrium

**Figure 5:**
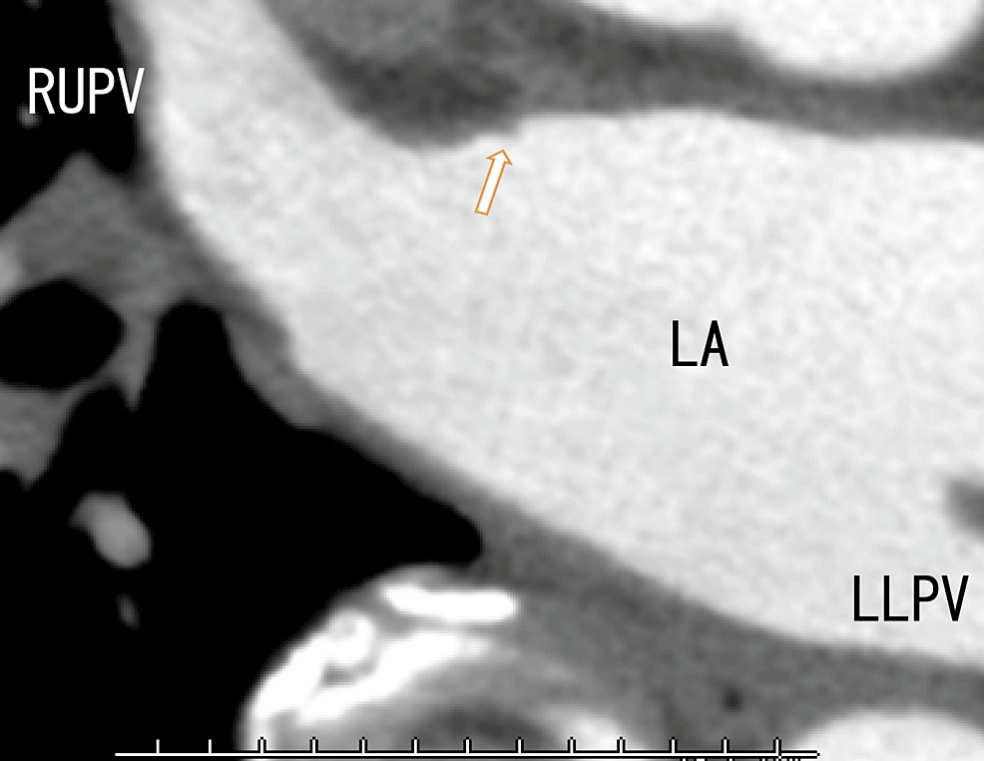
Axial 80-MDCT images showing a defect in the LA anterior wall around the entrance of the RUPV in the LA No thrombi were detected in the RUPV, and this slice is shown below Figure 3, suggesting that the thrombi in the RUPV are located anterosuperior to the RUPV. LA: left atrium; LLPV: left lower pulmonary vein; RUPV: right upper pulmonary vein

A cardiac CT revealed thrombi in the RUPV with a clear margin. We detected an LAD and collateral vessel near the LAD (Figures 3, 4) and a defect in the enhancement of the anterior wall of the LA (Figure 5), which should be near the LAD formation areas or the attachment areas of the LA thrombi expanded from the RLPV.

**Figure 6:**
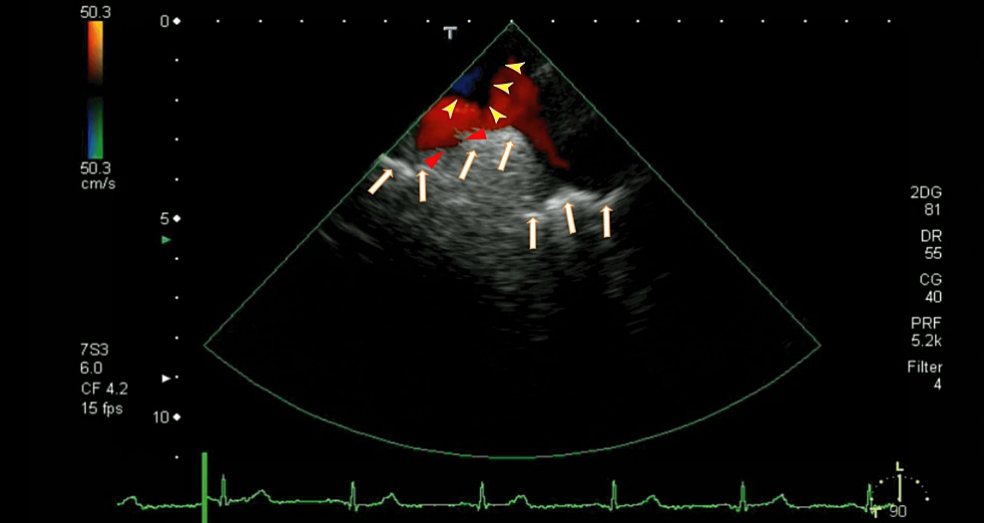
After three months of edoxaban treatment, TEE images showing thrombi in the LA Almost everything is similar to Figure 2. However, the dark thrombi identified by the blood flow from the RUPV seemed to disappear. The dark thrombi in front of the ostia of the right lower pulmonary vein (RLPV) remained, as illustrated by a lack of red blood flow (yellow arrowheads). Whole white regions seemed to become rather darker (arrows). LA: left atrium; LUPV: left upper pulmonary vein; RUPV: right upper pulmonary vein

To resolve the thrombus, edoxaban (30 mg, once daily) treatment was started. After three months of edoxaban treatment, TEE and cardiac CT were performed to clarify the changes in the above issues. TEE demonstrated that the white thrombi became rather dark and that there were fewer whitish thrombi around the ostia of the RLPV, indicating that edoxaban partially resolved the thrombi (Figure 6). The white thrombi on the anterior wall between the LUPV and the RUPV became rather darker, which was illustrated as a first stage, and moved like a frog-mouth, connecting the thrombi in the RLPV (Video 2).

**Video 2:** The video in Figure 6 Almost everything is similar to that of Video 1. ER and an end-QRS notch could not be observed on ECG at all.

**Figure 7:**
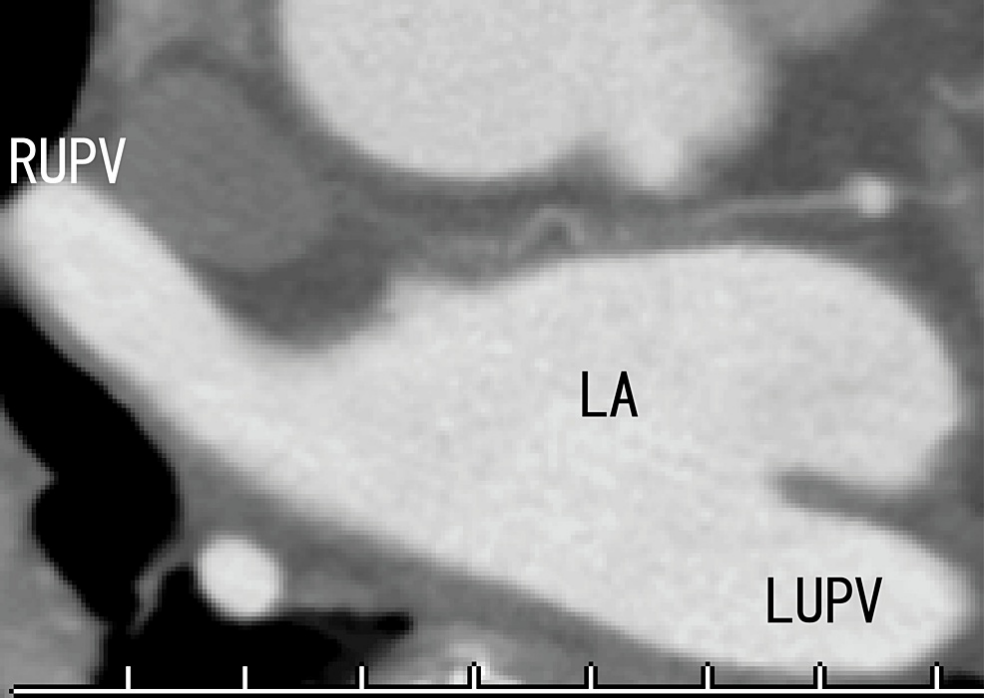
After three months of edoxaban treatment, axial 80-MDCT images revealed no thrombi in the RUPV Most parts of the images were similar to the images in Figure 3. RUPV: right upper pulmonary vein

**Figure 8:**
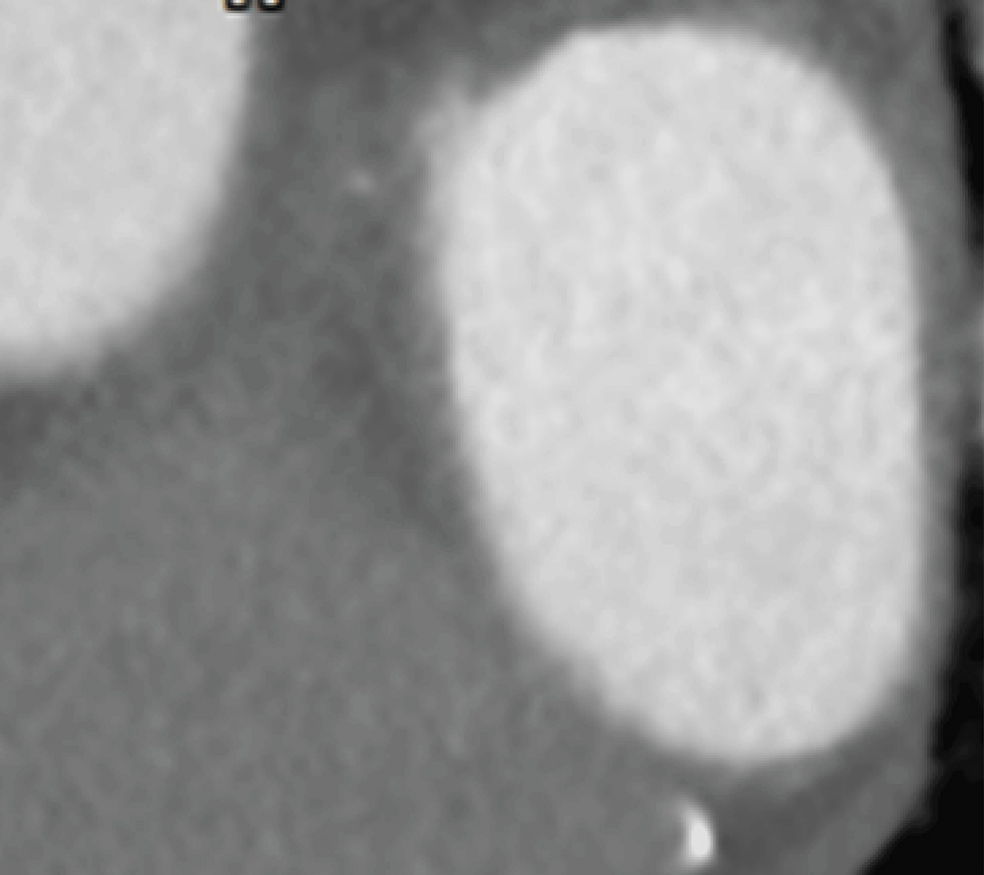
After three months of edoxaban treatment, sagittal images obtained via 80-MDCT revealed the LAD The size of the LAD seemed to increase slightly. LAD: left atrium diverticula

The 80-MDCT revealed no thrombus in the RUPV, indicating that the thrombi had resolved and that the LAD had become larger (Figures 7, 8). During this three-month treatment, the patient had no dizziness.

After nine months, anticoagulant therapy was continued. After three months, clopidogril was administered, and after six months, edoxaban (7.5 mg, once daily) treatment was continued.

**Figure 9:**
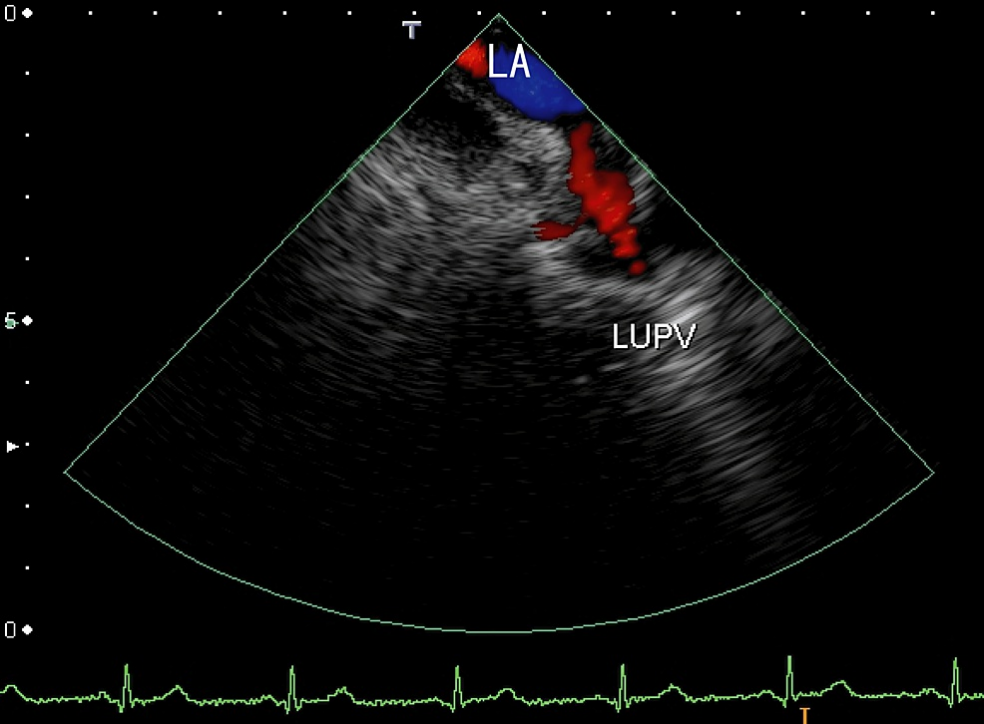
After the next nine months of anticoagulant treatment, TEE revealed no thrombi in the LA around the ostia of the LUPV LA: left atrium; LUPV: left upper pulmonary vein; TEE: transesophageal echocardiography

Nine months later, TEE revealed no white thrombi between the RUPV and LUPV, including frog mouth-shaped thrombi, indicating that those thrombi had resolved (Figure 9 and Video 3).

**Video 3:** The video in Figure 9 The white thrombi in the LA disappeared. LA: left atrium

**Figure 10:**
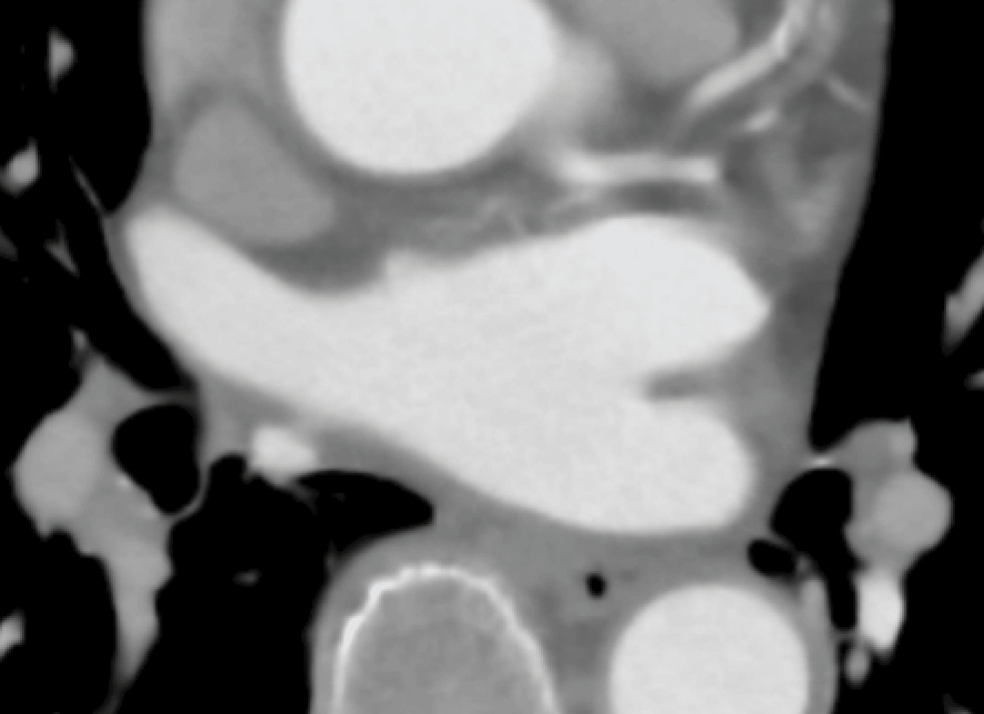
After nine months of anticoagulant treatment, axial 80-MDCT images showed no thrombi in the RUPV Most parts of the images were similar to the images in Figure 3 and Figure 7. RUPV: right upper pulmonary vein

**Figure 11:**
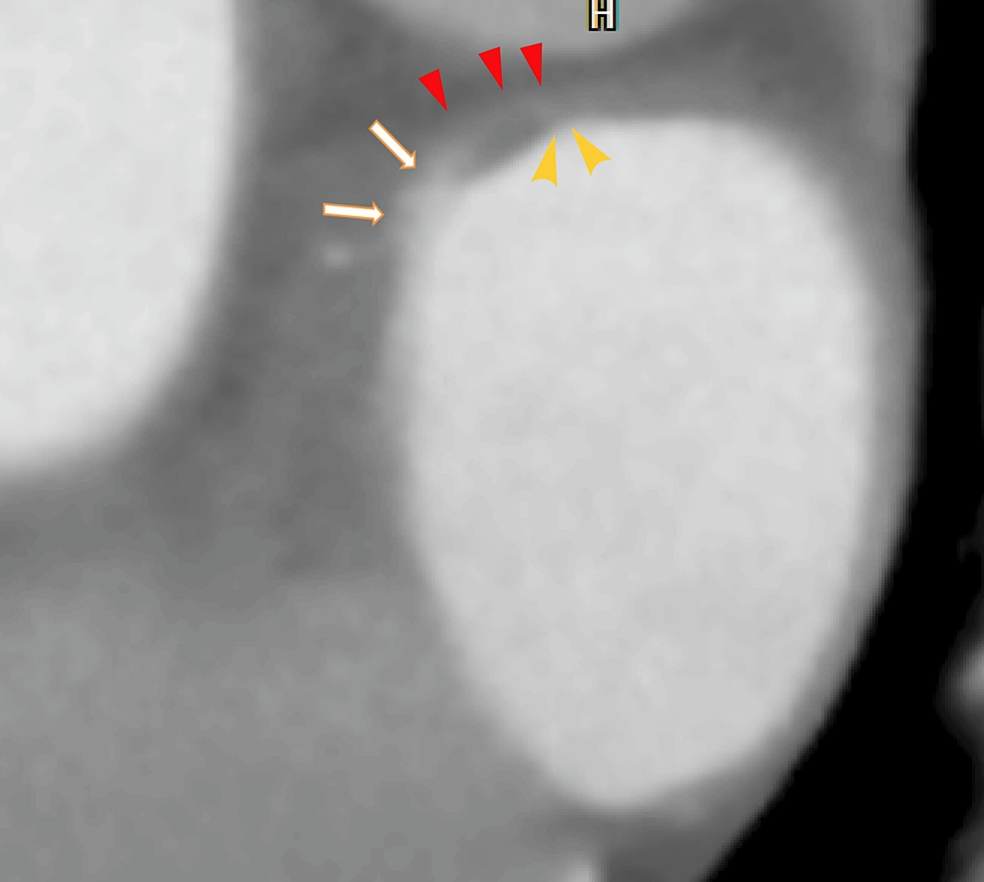
After the next nine months of anticoagulant treatment, sagittal images obtained via 80-MDCT revealed that the LAD had an annular shape Sagittal images of 80-MDCT showing the LAD on the LA anterior wall, which appeared to extend in the posterior direction (red arrowheads) and seemed to contact the LA superior wall (yellow arrows) with a black oval inside. LAD: Left atrium diverticula; LA: left atrium

**Figure 12:**
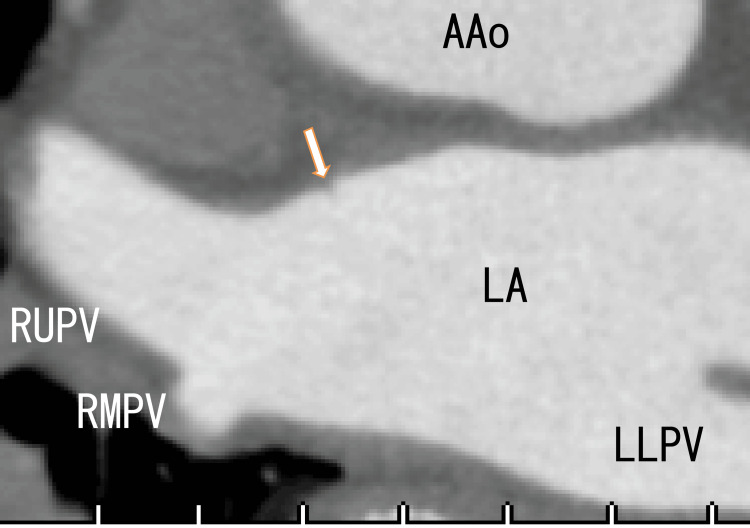
After one year of treatment, axial 80-MDCT images revealed a defect in the LA anterior wall around the entrance of the RUPV in the LA The shape was similar to that of Figure 4. AAo: ascending aorta; LA: left atrium; LLPV: left lower pulmonary vein; RLPV: right lower pulmonary vein; RUPV: right upper pulmonary vein

The 80-MDCT revealed no thrombus in the RUPV (Figure 10); however, the ends of the LAD and LA anterior walls seemed to be connected to vague white regions, including black ovals (Figure 11), which appeared circular. The defect of enhancement on the anterior wall of the LA remained, which seemed to become slightly smaller (Figure 12).

The defect of enhancement on the anterior wall of the LA remained, which seemed to become lightly smaller (Figure 12).

**Figure 13:**
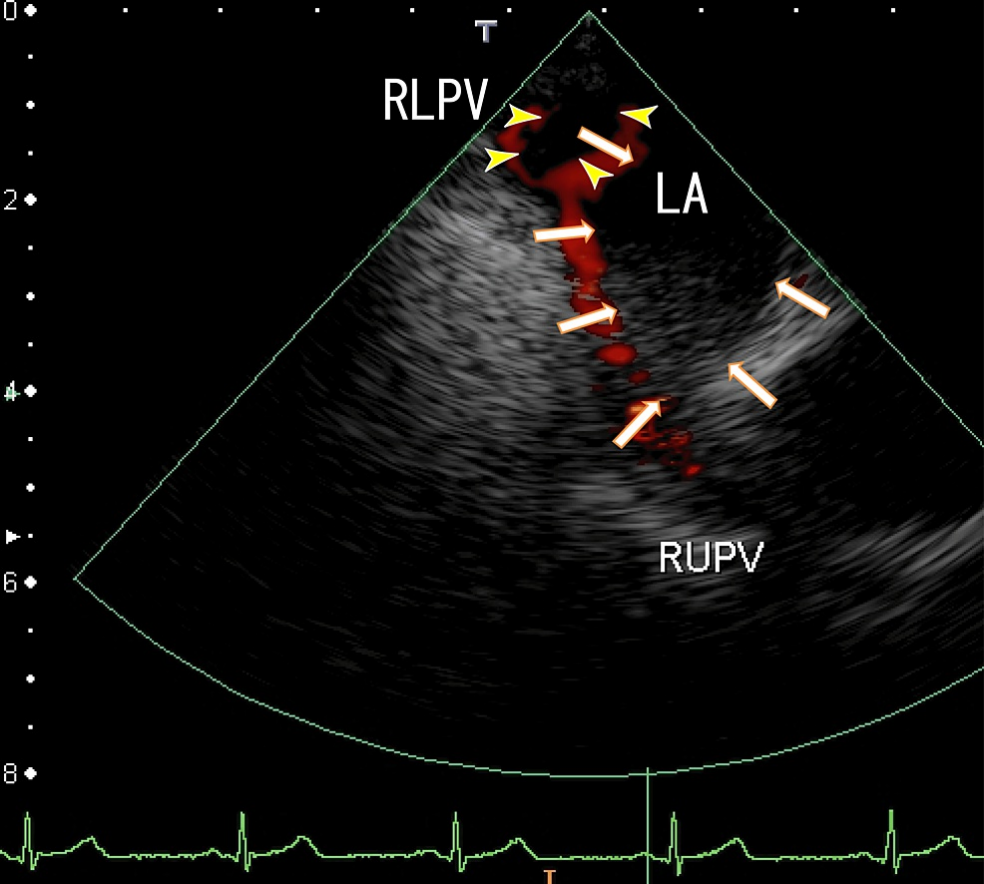
After the next nine months of anticoagulant treatment, TEE revealed two black thrombi in the LA around the ostia of the RUPV and RLPV Dark large thrombi emerged from the RUPV to the LA, so blood flow from the RUPV was limited. Dark small thrombi emerged around the ostia of the RLPV. LA: left atrium; RLPV: right lower pulmonary vein; RUPV: right upper pulmonary vein

Additionally, TEE images revealed large dark thrombi from the RUPV to the LA and small dark thrombi near the ostia of the RLPV (Figure 13 and Video 4).

**Video 4:** The video in Figure 13 Two dark thrombi were detected.

## Discussion

To our knowledge, this is the first report showing that collaterals from the left circumflex branch are connected to the anterior wall of the LA. We have previously reported that collaterals from the right coronary artery are connected to the LAD on the anterior wall of the LA [8].

The present case showed that there was a defect in the enhancement of the anterior wall of the LA, which might be relevant to some kind of small mass of the LA wall. To our knowledge, this is the first study to indicate this mechanism. A small defect in the anterior wall of the LA was found near the attachment regions of the LA thrombi. The defect was not affected by edoxaban therapy.

This is the first report to show that edoxaban ameliorates the presence of neither the notch nor the ER on the end-QRS. The ER and an end-QRS notch were observed first (Figure 2 and Video 1); however, after three months of edoxaban treatment, they were not observed (Figure 6 and Video 2). ER and end-QRS notches are associated with sudden cardiac arrest [15] and atrial fibrillation; however, how to treat these conditions is unclear. Edoxaban may have the potential to treat ER and end-QRS notch.

This is the first report to show that under white thrombus-resolving conditions, large dark thrombi formed around the ostia of the RUPV using TEE. In the present case, the whole thrombi in the RUPV were dissolved; however, this is uncommon. Our previous case reports showed that PVTs are usually not depicted with a clear margin using 80-MDCT and are partially resolved using warfarin and DAOCs [4]. The traits of the PVTs with clear margins, as assessed using 80-MDCT, were different from those of the PVTs without clear margins. PVTs with clear margins could be resolved with edoxaban as a whole.

Research on arterial thrombi (ATs) has shown that there are two types of ATs: white clots and red clots. White clots mainly consist of platelets and fibrin, whereas red clots mainly consist of red blood cells (RBCs). White clots from patients with ST-segment-elevation MI (STEMI) who are resistant to fibrinolysis are characterized by dense fibrin and increased contents of platelets, leukocytes, extracellular DNA, and von Willebrand factor (vWF) [17].

We believe that the white thrombi in the LA (Figure 2) were white clots, as estimated using TEE, so it is difficult to resolve them. The round thrombi around the ostia of the RLPV were red clots, so after three months, they partially resolved.

The third thrombi were the dark, rather round thrombi situated near the ostia of the RUPV, which emerged after three months of clopidogrel and nine months of a decreased dose of edoxaban. Cholesterol crystals are reportedly involved in the formation of retrieved thrombi, which are thought to be derived from cholesterol plaques [18].

The third might be cholesterol crystals, which have no or little fibrin, so the thrombi became round and larger despite fibrinolysis therapy. To our knowledge, this is the first study in which these characteristics of thrombi were assessed using TEE.

The present patient had an abnormally high level of protein S activity, which may be associated with platelet- and fibrin-poor thrombus formation and cholesterol crystals because protein S deficiency is related to platelet- and fibrin-rich thrombus formation [19]. Moreover, despite the use of a standard dose of edoxaban, thrombus formation has been reported in patients with protein S deficiency [20]. Moreover, abnormal protein S levels are likely to cause thrombi to form.

Recently, ozon contamination was reported to be associated with atherosclerosis via NET production [11]. The mechanisms underlying the linkage between air pollution and arteries are poorly understood. Dirty air might directly affect PVTs, especially neutrophils on PVTs, which may be linked to NET production associated with atherosclerosis. To clarify these relationships, more research is needed.

## Conclusions

Three months of standard-dose edoxaban treatment ameliorated the end-QRS notch and ER. The end of the LAD on the anterior wall of the LA extends in the posterior direction and vaguely touches the superior wall of the LA. Edoxaban resolved the existing white LA thrombi; however, new large dark thrombi appeared around the ostia of the RUPV.

## References

[1] Aspegren O, Staessens S, Vandelanotte S (2022). Unusual histopathological findings in mechanically removed stroke thrombi - a multicenter experience. Front Neurol.

[2] Saghamanesh S, Dumitriu LaGrange D, Reymond P, Wanke I, Lövblad KO, Neels A, Zboray R (2022). Non contrast enhanced volumetric histology of blood clots through high resolution propagation-based X-ray microtomography. Sci Rep.

[3] Takeuchi H (2022). Large arterial thrombi in the pulmonary vein are common in elderly subjects and may cause age-related disease by producing neutrophil extracellular traps. Cardiovascular Research.

[4] Takeuchi H (2014). High prevalence of pulmonary vein thrombi in elderly patients with chest pain, which has relationships with aging associated diseases. IJC Heart & Vessels.

[5] Sonobe S, Yoshida M, Niizuma K, Tominaga T (2019). Mechanical thrombectomy for acute ischemic stroke arising from thrombus of the left superior pulmonary vein stump after left pneumonectomy: a case report. NMC Case Rep J.

[6] Tsuji Y, Yagi R, Hiramatsu R, Wanibuchi M (2022). Mechanical thrombectomy for acute ischemic stroke due to thrombus in the pulmonary vein stump after left pulmonary lobectomy: a case series. Neurointervention.

[7] Takeuchi H (2024). Left atrial diverticula present in the right lower pulmonary vein thrombus attachment area. Cureus.

[8] Takeuchi H (2024). Left atrial diverticula supplied by the anomalistic branch of the right coronary artery. Cureus.

[9] Takeuchi H (2015). Nearly all left atrial thrombi may be extended from pulmonary vein thrombi. Int J Cardiol Heart Vasc.

[10] Shafqat A, Abdul Rab S, Ammar O (2022). Emerging role of neutrophil extracellular traps in the complications of diabetes mellitus. Front Med (Lausanne).

[11] Artner T, Lang IM (2024). From a dirty breath to atherosclerosis: ozone pollution and neutrophil extracellular traps (NETs). Atherosclerosis.

[12] Blasco A, Rosell A, Castejón R (2024). Analysis of NETs (neutrophil extracellular traps) in coronary thrombus and peripheral blood of patients with ST-segment elevation myocardial infarction. Thromb Res.

[13] Dumont BL, Neagoe PE, Charles E (2024). Low density neutrophils and neutrophil extracellular traps (NETs) are new inflammatory players in heart failure. Can J Cardiol.

[14] Şeker M (2020). The characteristics of left atrial diverticula in normal sinüs rhythm patients. Surg Radiol Anat.

[15] Pieroni M, Bellocci F, Crea F (2008). Sudden cardiac arrest associated with early repolarization. N Engl J Med.

[16] Hasegawa Y, Sugiura H, Sanada A (2024). Association between early repolarization and vagally mediated atrial fibrillation. Heart Rhythm.

[17] Staessens S, Denorme F, Francois O (2020). Structural analysis of ischemic stroke thrombi: histological indications for therapy resistance. Haematologica.

[18] Silvain J, Collet JP, Nagaswami C (2011). Composition of coronary thrombus in acute myocardial infarction. J Am Coll Cardiol.

[19] Brouns SL, Tullemans BM, Bulato C (2022). Protein C or Protein S deficiency associates with paradoxically impaired platelet-dependent thrombus and fibrin formation under flow. Res Pract Thromb Haemost.

[20] Lee WC, Huang MP (2021). Lead thrombus under standard-dose edoxaban in a patient with normal to high creatinine clearance and protein S deficiency. Thromb J.

